# Free-Radical Propagation Rate Coefficients of Diethyl Itaconate and Di-n-Propyl Itaconate Obtained via PLP–SEC

**DOI:** 10.3390/polym15061345

**Published:** 2023-03-08

**Authors:** Enno Meyer, Tobias Weege, Philipp Vana

**Affiliations:** Institut für Physikalische Chemie, Georg-August-Universität Göttingen, Tammannstraße 6, 37077 Göttingen, Germany

**Keywords:** propagation rate coefficient, *k*_p_, PLP-SEC, itaconate, kinetics, radical polymerization

## Abstract

The propagation step is one of the key reactions in radical polymerization and knowledge about its kinetics is often vital for understanding and designing polymerization processes leading to new materials or optimizing technical processes. Arrhenius expressions for the propagation step in free-radical polymerization of diethyl itaconate (DEI) as well as di-n-propyl itaconate (DnPI) in bulk, for which propagation kinetics was yet unexplored, were thus determined via pulsed-laser polymerization in conjunction with size-exclusion chromatography (PLP-SEC) experiments in the temperature range of 20 to 70 °C. For DEI, the experimental data was complemented by quantum chemical calculation. The obtained Arrhenius parameters are *A* = 1.1 L·mol^–1^·s^–1^ and *E*_a_ = 17.5 kJ·mol^−1^ for DEI and *A* = 1.0 L·mol^–1^·s^–1^ and *E*_a_ = 17.5 kJ·mol^−1^ for DnPI.

## 1. Introduction

Esters of itaconic acid (2-methylenesuccinic acid) show the potential to replace styrene as a comonomer in the synthesis of polymer resins [1] or as composite material with cotton [2]. This is especially interesting given the fact that itaconic acid can be produced by many microbiological organisms such as *Aspergillus terreus* and thus can be obtained from biomass while styrene is petrol based [3,4,5].

In order to fully utilize itaconic acid esters in free-radical polymerizations, the exact knowledge of the reaction rate coefficients is highly advantageous. Here, we are focusing on the propagation rate coefficient, $k_{p}$. While in a number of publications [6,7,8,9,10,11,12,13], data for $k_{p}$ for a few members of the series of itaconic acid homo-diesters are already available, this series is lacking some data, most notably for the diethyl and the di-n-propyl ester. This work intends to fill these gaps by applying pulsed-laser polymerization in conjunction with the size-exclusion chromatography (PLP-SEC) method as well as quantum chemical prediction in order to obtain Arrhenius expressions for $k_{p}$ of these monomers.

PLP is a highly specialized technique for studying the kinetics of radical polymerization reactions. It involves using a pulsed laser to initiate polymerization in short bursts, allowing for precise control over reaction conditions and reaction kinetics. One of the key advantages of PLP is that it allows for the measurement of propagation rate coefficients of radical polymerization with high precision and accuracy, and is thus IUPAC-recommended. The propagation rate coefficient, *k*_p_, is one of the kinetic key parameters that describe how fast the chain-growth reaction proceeds and how sensitive it is to changes in reaction conditions such as temperature and concentration of reactants. Measuring kinetic coefficients in radical polymerization is important for understanding and optimizing radical polymerization processes, as it allows to predict and control the behavior of the reaction under different conditions and in addition enables to predict the properties of the resulting polymer. The kinetic information can thus be used to design new polymers with specific properties, optimize reaction conditions for industrial-scale production, and troubleshoot problems that arise during polymerization.

## 2. Materials and Methods

Chemicals: Diethyl itaconate (DEI, *M* = 186.21 g/mol) (98%) was purchased from Tokyo Chemical Industry (TCI). The inhibitor 4-tert-butylcatechol (TBC) was removed via column chromatography over alkaline aluminum oxide.

Di-n-propyl itaconate (DnPI, *M* = 214.26 g/mol) was obtained by esterification of itaconic acid (TCI, 99%) with 1-propanol (Sigma-Aldrich, 99.5%) according to a literature procedure [14].1 eq. of itaconic acid was dissolved in 10 eq. of 1-propanol and acidified with 0.4 eq. conc. H_2_SO_4_. The mixture was boiled under reflux for 5 h. Then, the alcohol was removed using a rotary evaporator. The residue was poured in a 5-times excess of ice water, the phases were separated, and the organic phase was neutralized with a 10 wt.%-solution of NaHCO_3_ in water. Afterwards, the product was dried over CaCl_2_. The purity was determined by NMR and was found to be >95 %.

**Scheme 1:**
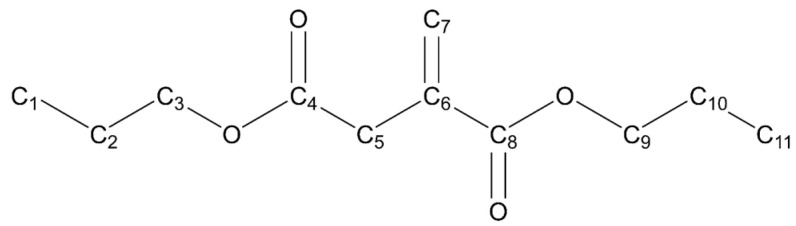
Carbon atom numbering for DnPI used in the NMR assignment.

^1^H NMR (300 MHz, CDCl_3_, δ, see Scheme 1): 6.31 (s, 1H; C7), 5.68 (s, 1H; C7), 4.11 (t, *J* = 6.8 Hz, 2H; C3), 4.04 (t, *J* = 6.8 Hz, 2H; C9), 3.33 (s, 2H; C5), 1.65 (m, 4H; C2+C10), 0.92 (q J = 7.3 Hz, 6H; C1+C11); ^13^C NMR (75 MHz, CDCl_3_, δ): 170.9 (C4), 166.4 (C8), 134.3 (C6), 128.2 (C7), 66.72 (C3/C9), 66.6 (C3/C9), 37.9 (C5), 22.06 (C2+C10), 10.5 (C1+C11).

The photoinitiator 2,2-dimethoxy-2-phenylacetophenone (DMPA, Aldrich, 99%) was used as received.

PLP experiments: All samples were degassed by a gentle flow of argon for at least 10 min. Pulsed-laser-polymerizations were performed in silica glass cells which were tempered by a heating bath for at least 10 min prior to the start of irradiation. The laser used for irradiation is an ATLEX 1000 I XeF exciplex laser operating at 351 nm with a pulse energy of 1–7 mJ. Repetition rates varied from 0.25 to 2 Hz. The samples were prepared by dissolving 20 mM to 75 mM of DMPA in the respective itaconate ester. No further solvent was added. 

After irradiation, the mixture was used directly for SEC analysis without isolation of the polymer.

Density measurements: A gas pycnometry system (AccuPyc II from Micromeritics) was used to measure room temperature densities of the monomers. Since the instrument did not have a temperature control system, room temperature densities were used for the whole temperature range. The following densities were obtained: 1.044 g·mL^−1^ (DEI) and 1.025 g·mL^−1^ (DnPI).

Size-exclusion chromatography: Size-exclusion chromatography was performed using an Agilent 1260 G1310B iso pump with a PSS degasser. Injection was performed with an Agilent 1260 ALS G1329B autosampler onto three PSS SDV 5 μm bead columns (10^6^,10^4^,10^3^ Angstrom) which was embedded in a PSS TCC6000 column oven at 35 °C. THF at a flow rate of 1 mL·min^−1^ was used as the solvent. The detector was an Agilent 1260 RID G1362A. Access to absolute molecular weight distributions was obtained by a PS-PMMA calibration in conjunction with the Mark-Houwink-constants of DEI and DnPI, which have been determined previously [15]. These values refer to the solvent toluene, but may also be used for THF, as suggested by Szablan et al. [7].

Quantum chemical calculations: A propagating DEI radical was mimicked by a DEI molecule with a methyl group bound to the former olefinic double bond. Minimum structures of the reactants (consisting of the radical and a monomer) and the product were found using the crest program by Grimme [16]. Repeated runs on different conformers of reactant and product were performed and the global minima were identified. These global minima were optimized with ORCA 4.2.1 and subsequently a NEB-TS-calculation was performed to find the TS structure [17,18].

## 3. Results

The Arrhenius parameters for the propagation step of the sterically hindered monomer DEI were calculated using quantum chemistry and transition state theory (TST). In order to obtain experimental values for $k_{p}$, PLP-SEC experiments were performed for DEI in bulk in the temperature range between 20 and 70 °C and for DnPI in the temperature range of 30 to 70 °C. For the basic principles of the IUPAC-recommended PLP-SEC method and for experimental peculiarities, please also refer to [19,20].

### 3.1. Prediction of Arrhenius Parameters for DEI

$k_{p}$ as function of temperature, *k*_p_(*T*), can be calculated from the partition functions $Q_{n}$ of the involved molecules n and the electronic barrier height $E_{0}$ (see Equation (1)). κ denotes a correction factor in order to account for tunneling (assumed to be equal to 1 here and will be left out in the following), c is the inverse of the volume used in the translational partition function, *k_B_* is the Boltzmann constant, *R* the universal gas constant, and m is the molecularity of the reaction [21].

(1) $$k_{p}\left(T\right)\;=\;\kappa \cdot \;c^{\left(1-m\right)}\cdot \frac{k_{B}T}{h}\cdot \frac{Q^{‡}}{\prod_{n}Q_{n}\;}\cdot \exp\left(-\frac{E_{0}}{RT}\right)$$

This expression can also be reformulated using the thermodynamical properties entropy of activation, $\Delta S^{‡}$, and enthalpy of activation, $\Delta H^{‡}$:

(2) $$k_{p}\left(T\right)=c^{\left(1-m\right)}\cdot \frac{k_{B}T}{h}\exp\left(\frac{\Delta S^{‡}}{R}\right)\cdot \exp\left(-\frac{\Delta H^{‡}}{RT}\right)$$

From Equation (2), the Arrhenius parameters for *k*_p_ can be calculated directly by Equations (3) and (4). The formulae for calculating the individual entropy components can be found in the Supporting Information.

(3) $$A=c^{\left(1-m\right)}\cdot \exp\left(m\right)\cdot \frac{k_{B}T}{h}\exp\left(\frac{\Delta S^{‡}}{R}\right)$$

(4) $$E_{a}=E_{0}+\mathrm{ZPVE}+\Delta \Delta H^{‡}+mRT$$

ZPVE is the vibrational zero-point energy and $\Delta \Delta H^{‡}$ a temperature correction, which is calculated for a single species by Equation (5) where ν_j_ denotes the frequency of the j-th vibration and *h* is the Planck constant.

(5) $$\Delta \Delta H=R\cdot \underset{j}{\sum }\frac{h\nu_{j}}{\exp\left(h\nu_{j}\right)-1}+\frac{5}{2}RT+\frac{3}{2}RT$$

Itaconates are 1,1-disubstituted ethylene derivatives and thus four different propagation variants are possible. The attacking radical as well as the resulting macroradical can each be centered on the tertiary or the primary carbon. Radical stability favors the radical to be centered on the tertiary carbon, while steric hindrance favors an attack on the primary carbon, resulting in the formed radical also to be tertiary. Consequently, only this reaction has been considered in this work. One might improve the results presented here by considering all variants and Boltzmann-weighing them.

Geometries and frequencies are rather insensitive to the level of theory, so using a cheap method is recommended. The electronic barrier, on the other side, is typically very sensitive to the level of theory used, so a high-quality method should be used. However, for reasons of computational power, in this work only UHF/6-31G(d) and B3LYP/def2-TZVP were used.

The calculations were carried out in the gas phase. More accurate results can be obtained by including a solvent model to include interactions between the solute and solvent. These can influence for example electronic energy levels and vibrational frequencies [22]. Since the monomer in question is rather exotic, no implicit solvent model parameters exist for it, to our best knowledge. Consequently, an explicit solvent model would have to be used. However, this comes at a largely increased computational cost and is far beyond the available resources. Consequently, no solvent model could be used.

The vibrational frequencies were used unscaled as well as scaled. For UHF/6-31G(d), the average of the scaling factors reported by Scott et al. [23] was used, which amounts to 0.90064. For B3LYP/def2-TZVP, the average of the scaling factors reported by Kesharwani et al. [24] was used, which amounts to 0.9870. 

The obtained minimum structures (on UHF/6-31G(d) level of theory) are depicted in the Appendix B and their .xyz-coordinates are also given. It is clearly visible that both molecules are approximately orthogonally aligned to each other in the reactant structure, in order to minimize steric repulsion. In the transition state (TS), however, both molecules are aligned in parallel, in order to facilitate overlap of the involved orbitals. The product structure is very similar to the TS structure, indicating a late TS. This is unexpected since exothermic reactions as these typically exhibit an early TS.

Obtained electronic barrier heights and Arrhenius parameters are shown in Table 1. The Arrhenius activation energy is very similar for both levels of theory. Likely, a high level of theory such as CCSD(T) would increase the accuracy significantly. The Arrhenius prefactors differ by approximately one order of magnitude for both levels of theory. Both, however, are significantly lower compared to other typical classes of monomers such as acrylates, where they are in the order of $10^{6}\;L\cdot {\mathrm{mol}}^{-1}\cdot s^{-1}$ [25]. This can be explained by the bulky substituents at the olefinic bond, which hinder the approach of a new monomer for reaction.

### 3.2. PLP Experiments

**Figure 1:**
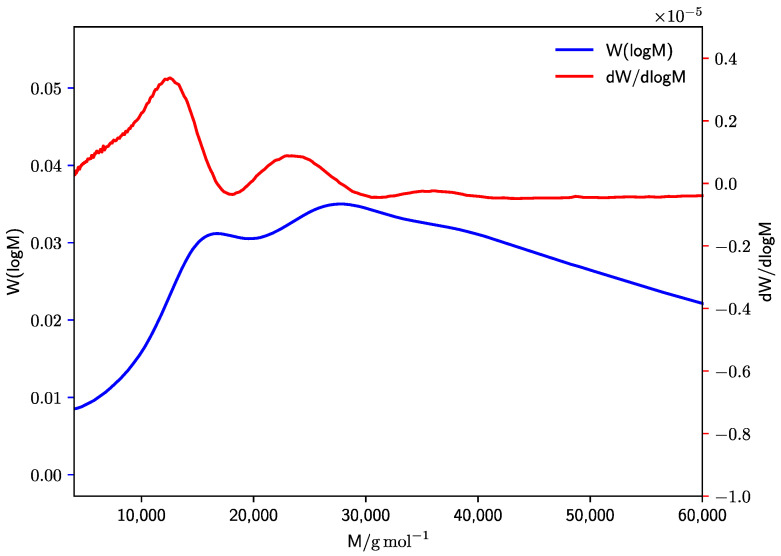
Detail of a representative molar mass distribution (**blue**) from PLP of DEI and its derivative (**red**). The molar masses *L*_i_ were obtained from the maxima of the derivative curve.

The PLP-SEC-method introduced by Olaj and coworkers [26] offers a very easy way to determine propagation rate coefficients in radical polymerization. A sample consisting of monomer, photoinitiator, and solvent (if applicable) is subjected to repeated, short laser pulses. The laser pulse initiates polymerization by instantaneous formation of radicals. In between the pulses, the radicals propagate (and also terminate to some extent) but upon the following laser pulse, the radical concentration rises sharply again, and the termination probability is thus greatly enhanced. The length L of the chains which were initiated by a laser pulse and were terminated in the moment of the following laser pulse corresponds to $L=k_{p}\cdot c_{M}\cdot t_{0}$ with $t_{0}$ being the time between two consecutive laser pulses and $c_{M}$ the monomer concentration. However, not all radicals terminate at the first following laser pulse, but may be stopped at the second or third (*i*-th) subsequent pulse. The corresponding chain length is then $L_{i}=i\cdot k_{p}\cdot c_{M}\cdot t_{0}$, provided the monomer conversion is kept relatively low. In any case, a polymer with that specific chain length of *L*, 2*L*, 3*L*, … is more frequently occurring than that having other chain lengths, and thus, structured chain-length distributions with multiple maxima are typically observed. The structure can be evaluated to obtain estimates for *L_i_* and the inflection point at the low molecular weight side of the peak typically yields the most precise results [26]. The inflection points were determined by evaluating the maxima of the derivative of the molar mass distribution, as shown in Figure 1.

Itaconates propagate slowly and also terminate slowly [8], which presents a challenge for the PLP method. In this work, we addressed this challenge by choosing an extremely slow laser repetition rate, which allowed us to obtain successful results.

#### 3.2.1. Diethyl Itaconate

The PLP-SEC data obtained for DEI are presented in Table 2. For each temperature, two separate solutions of the photoinitiator 2,2-dimethoxy-2-phenylacetophenone (DMPA) in DEI were prepared and from each solution two experiments were performed. The molar masses at the inflection points, *M*_Inf_, were extracted from complete molar mass distributions obtained via size-exclusion chromatography.

To verify the integrity of the data, the obtained values of $k_{p}$ shall be independent of the initiator concentration and the repetition rate. Furthermore, the position of the second additional PLP peak shall be at double the molar mass of the first peak and analogously the third peak at triple the molar mass of the first peak (or 1.5 times of the second peak). This requirement rests on the assumption that *k*_p_ is chain-length independent and is called the “consistency criterion” of PLP-SEC. It was introduced for distinguishing the additional PLP peaks from any other occurring peaks that are not kinetically relevant.

**Figure 2:**
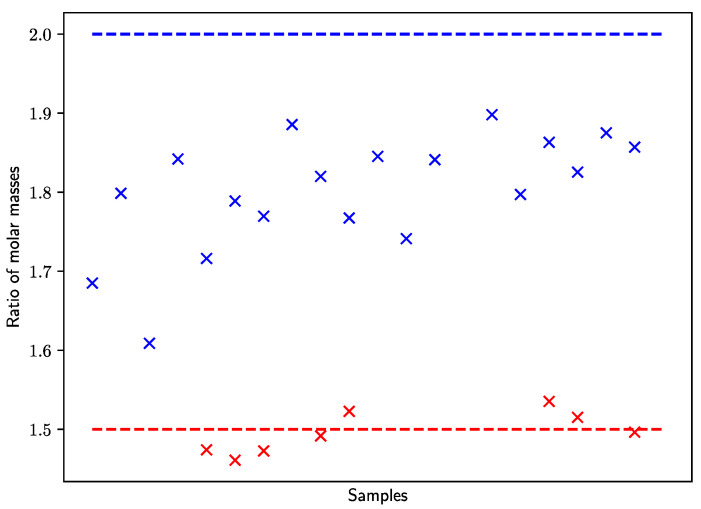
Ratio of molar masses of inflection points for all DEI samples. Blue points show the ratio of the second inflection point to the first one, while the red points show the ratio of the third inflection point to the second one.

As can be seen in Figure 2, the ratio of the second peak position divided by the first is systematically lower than 2, indicating a significant chain length dependence of propagation for chain lengths up to ca. 150 to 200. For the ratio between the third and second peak, however, the agreement with the expected ratio of 1.5 is good. This observation has been made for many other systems studied via PLP-SEC [27,28,29,30] and there still is discussion, whether this effect—which is clearly going beyond the generally accepted chain length dependency of *k*_p_ for short macroradicals up to chain lengths of ca. 5 [31,32]—is reflecting a real chain-length dependency up to longer chain-lengths or is an SEC artifact [33,34]. PLP-SEC alone is apparently not capable of deciding this open question and it is now generally accepted that the *k*_p_ data obtained are estimates subject to uncertainty, due to such as yet unexplained effects.

**Figure 3:**
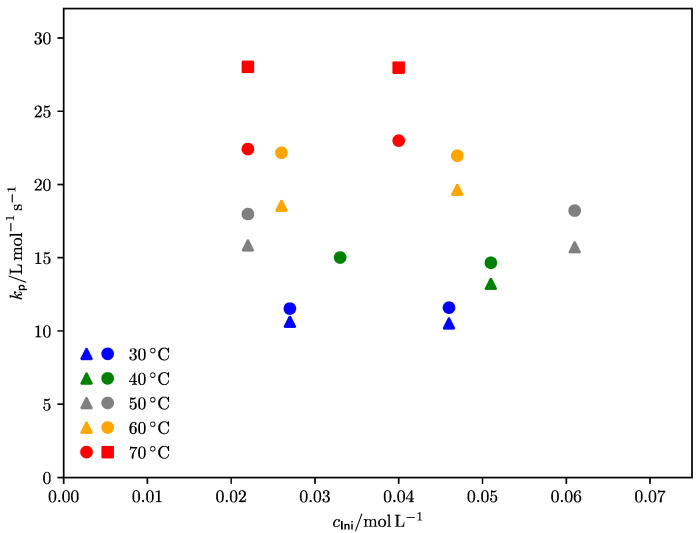
Obtained propagation rate coefficients, *k*_p_, for DEI as function of initiator concentration, *c*_Ini_. An average of the $k_{p}$ values extracted from the different inflection points is used here. Triangles refer to a laser repetition rate of 0.5 Hz, while circles refer to 1 Hz, and squares to 2 Hz.

Figure 3 shows the obtained $k_{p}$ values for different temperatures and different laser pulse frequencies as a function of initiator concentration. It is clearly visible that $k_{p}$ is independent of initiator concentration—a strict criterion for a successful PLP-SEC experiment –, but, however, is not independent of the repetition rate, which alters the chain length of the produced polymer, as discussed above. With increasing repetition rate, $k_{p}$ increases. 

**Figure 4:**
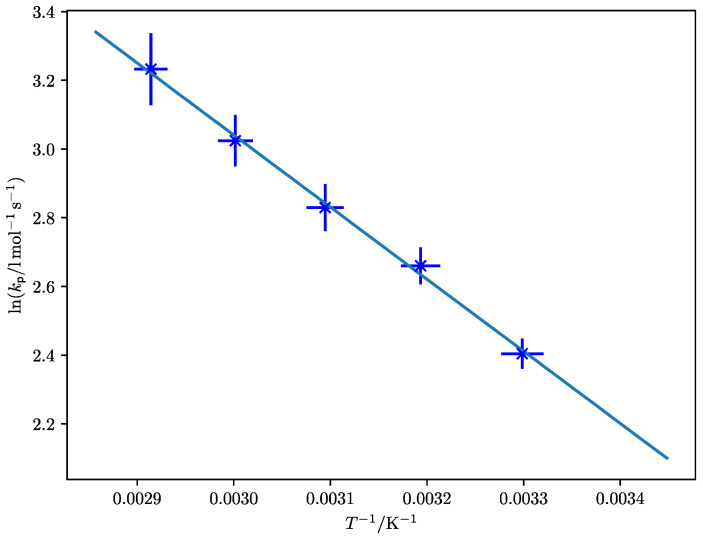
Arrhenius fit for $k_{p}$ of DEI. The indicated uncertainties in ln(*k*_p_) result from the standard deviation of the measurements for the respective temperature, while for the uncertainty in *T*^−1^ an uncertainty of Δ*T* = 2 K was assumed. The obtained Arrhenius parameters are shown in Table 3.

In order to extract the Arrhenius parameters from the obtained *k*_p_ data, a regular Arrhenius fit is employed (see Figure 4). The observed chain-length dependency of *k*_p_ is not taken into account, but is accepted as uncertainty; consequently, the Arrhenius parameters, given in Table 3, will be average values over the whole chain length regime. It can already be noted here that these Arrhenius parameters fit very well to the ones obtained for other itaconates, but this will be discussed in greater detail below. 

#### 3.2.2. Di-n-Propyl Itaconate

Other than for DEI, only two stock solutions of DnPI with different concentrations of DMPA were prepared. The pulse energy was not recorded explicitly during the irradiation but was in a similar range as for the DEI experiments. For all samples, 1000 pulses were irradiated onto the sample, guaranteeing that the overall monomer conversion was low, as required for a successful PLP-SEC-experiment. The resulting PLP-data are collated in Table 4.

**Figure 5:**
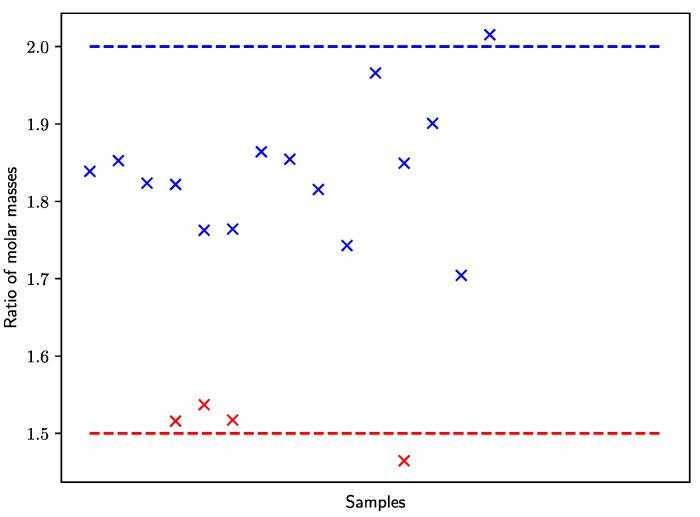
Ratio of molar masses of inflection points for all DnPI samples. Blue points show the ratio of the second inflection point to the first one, while the red points show the ratio of the third inflection point to the second one. Data points that significantly deviate from the expected ratios have been omitted from the analysis according to the consistency criterion of PLP-SEC.

The same consistency criteria as for DEI were checked (see Figure 5). When inspecting the ratio of molar masses of the inflection points, clearly a higher scattering is observed compared to DEI. The data for the ratio of peaks 2 and 1 also shows that most samples are well below the anticipated value of 2, again pointing towards a chain-length dependency of $k_{p}$.

**Figure 6:**
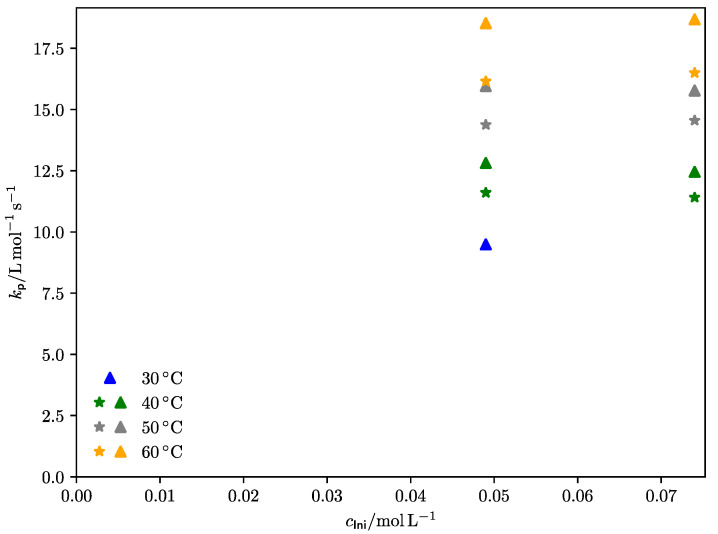
Obtained propagation rate coefficients for DnPI as a function of initiator concentration, *c*_Ini_. An average of the $k_{p}$ values extracted from the different inflection points is used here. Stars refer to a laser repetition rate of 0.25 Hz, while triangles refer to 0.5 Hz.

As can be seen in Figure 6, just as for DEI, the obtained $k_{p}$ values are independent of initiator concentration and increase with increasing laser repetition rate. The same reasoning as for DEI can be applied here.

**Figure 7:**
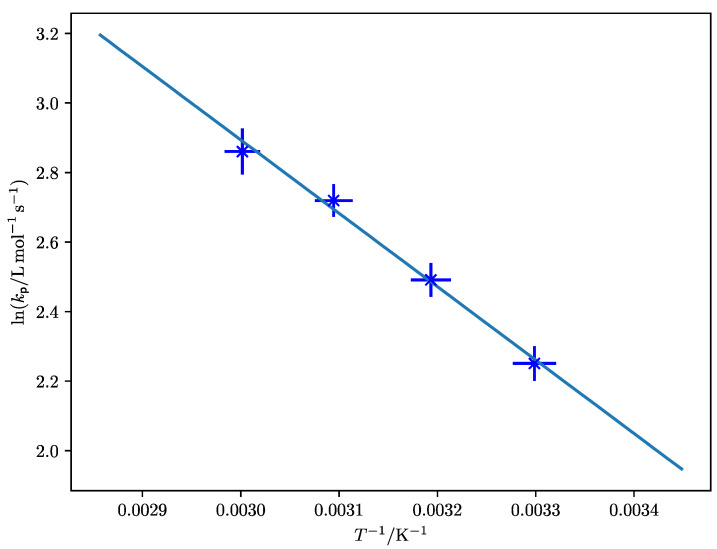
Arrhenius fit for $k_{p}$ of DEI. The indicated uncertainties in ln(*k*_p_) result from the standard deviation of the measurements for the respective temperature, while for the uncertainty in *T*^−1^ an uncertainty of Δ*T* = 2 K was assumed. For 30 °C, only one data point exists, consequently the uncertainty in ln(*k*_p_) was assumed to be similar to the other values. The obtained Arrhenius parameters are shown in Table 5.

Since for 30 °C only one data point exists, the uncertainty for the Arrhenius fit, which is shown in Figure 7, was assumed to be similar to that of the other data points.

## 4. Discussion

Inspecting the experimental results of the Arrhenius fits, one notices that the results for DEI and DnPI are extremely close to each other. A correlation analysis for the Arrhenius parameters has not been performed, however. Within the series of itaconate esters (see Table 6), DEI and DnPI are on the lower end of prefactor and activation energy values.

The quantum chemical calculations show good agreement with the experimental results. Compared to the experimental values, the prefactors match nearly perfectly on B3LYP/def2-TZVP level. The predicted activation energy is about 5 kJ/mol too high which is still a very good agreement, considering the rather low level of theory. This good agreement is likely due to error compensation.

**Figure 8:**
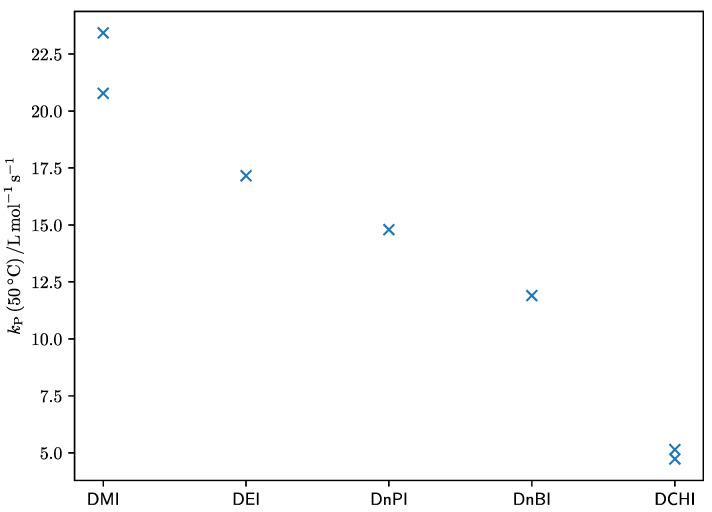
Values of *k*_p_ at 50 °C for the family of itaconates using Arrhenius parameters from Table 6.

Haehnel et al. [35] investigated the family behavior of linear alkyl methacrylate monomers. They stated that high frequency factors are accompanied by high values of the activation energy, making it difficult to identify any trends with Arrhenius parameters. Therefore, the actual values of *k*_p_ at a given temperature were used for revealing family behavior of monomers. In the case of linear alkyl methacrylates, *k*_p_ (at 50 °C) increases linearly with the length of the side chain [34]. For itaconates, we find an opposite trend: as indicated in Figure 8, *k*_p_ clearly decreases with increasing size of the ester groups. This can possibly be reasoned by a pre-structuring effect of the monomer, as has also been put forward by Haehnel et al. [35]. For alkyl methacrylates, longer side chains lead to a more structured monomer bulk. Due to stronger dispersive interactions, the longer side chains align more pronouncedly with each other. This effect also aligns the olefinic bonds such that propagation is easily possible and promoted with ester groups size. Itaconates, however, have two side chains. This allows for two different patterns of alignment. The first is identical to the one discussed for methacrylates. The second pattern is not stacked, but shifted, in a zig-zag motif. This alignment increases the distance of the olefinic bonds, leading to decreased propagation rates. Apparently for itaconates, the second pattern is preferred. This interesting effect might be confirmed by MD simulations in the future.

## 5. Conclusions

Arrhenius expressions for the propagation step in free-radical polymerization of diethyl itaconate (DEI) as well as di-n-propyl itaconate (DnPI) in bulk, for which propagation kinetics was yet unexplored, were determined via pulsed-laser polymerization in conjunction with size-exclusion chromatography (PLP-SEC) experiments in the temperature range of 20 to 70 °C. The values were found to be very similar for both monomers. Together with literature data for other itaconate monomers, a family behavior was found, where *k*_p_ clearly decreases with increasing size of the ester groups. This behavior is different to other monomers and can possibly be explained by a pre-structuring effect of these rather sterically hindered monomers.

## Figures and Tables

**Table 1:** Calculated electronic barriers, *E*_0_, and Arrhenius parameters (prefactor, *A*, and activation energy, *E_A_*) for the propagation step of DEI at 20 °C. The frequencies used for the calculation were used unscaled as well as scaled by their respective scaling factors.

Method	*E*_0_ [kJ·mol^–1^]	*A* [L·mol^–1^·s^–1^]	*E_A_* [kJ·mol^–1^]
UHF/6-31G(d)	12.7	$2.0\cdot 10^{3}$	22.6
UHF/6-31G(d) scaled	12.7	$3.3\cdot 10^{3}$	22.3
B3LYP/def2-TZVP	13.8	$1.4\cdot 10^{4}$	22.7
B3LYP/def2-TZVP scaled	13.8	$1.5\cdot 10^{4}$	22.7

**Table 2:** PLP-SEC data for DEI: initiator concentration, *c*_Ini_, temperature *T*, total number of pulses, *n*_pulses_, laser pulse frequency, *ν*, molecular weight at the various inflection points, *M*_inf_, and the average *k*_p_ of all obtained inflection points.

*c*_Ini_ [mol·L^–1^]	*T*/K	*n*_pulses_	*ν* [Hz]	*M*_inf1_ [g·mol^–1^]	*M*_inf2_ [g·mol^–1^]	*M*_inf3_ [g·mol^–1^]	*k*_p_ [L·mol^–1^·s^–1^]
0.022	343.15	2000	1	25,400	42,800	-	22.4
0.022	343.15	2000	2	15,400	27,700	-	28.0
0.040	343.15	2000	1	26,600	42,800	-	23.0
0.040	343.15	2000	2	15,200	28,000	-	28.0
0.026	333.15	1000	0.5	43,000	73,800	108,800	18.5
0.026	333.15	4000	1	25,100	44,900	65,600	22.2
0.047	333.15	2400	0.5	44,700	79,100	116,500	19.7
0.047	333.15	4000	1	23,600	44,500	-	22.0
0.022	323.15	2000	1	20,000	36,400	54,300	18.0
0.022	323.15	2000	0.5	35,700	63,100	96,100	15.9
0.061	323.15	2000	1	19,780	36,400	-	18.2
0.061	323.15	2000	0.5	35,100	61,120	-	15.7
0.033	313.15	2000	1	16,320	30,050	-	15.0
0.051	313.15	2000	1	15,700	29,800	-	14.7
0.051	313.15	2000	0.5	29,100	52,300	-	13.2
0.027	303.15	2000	1	12,510	23,310	35,790	11.5
0.027	303.15	2000	0.5	23,500	42,900	65,000	10.6
0.046	303.15	2000	1	12,490	23,420	-	11.6
0.046	303.15	2000	0.5	23,100	42,900	64,200	10.5

**Table 3:** Experimental Arrhenius parameters for chain-length-averaged *k*_p_ of DEI, obtained via PLP-SEC.

*A* [L·mol^–1^·s^–1^]	*E_A_* [kJ·mol^–1^]
(1.1 ± 0.3) · 10^4^	17.5 ± 0.6

**Table 4:** PLP data for DnPI: initiator concentration, *c*_Ini_, temperature *T*, total number of pulses, *n*_pulses_, laser pulse frequency, *ν*, molecular weight at the various inflection points, *M*_inf_, and the average *k*_p_ of all obtained inflection points.

*c*_Ini_ [mol·L^–1^]	*T*/K	*ν* [Hz]	*M*_inf1_ [g·mol^–1^]	*M*_inf2_ [g·mol^–1^]	*M*_inf3_ [g·mol^–1^]	*k*_p_ [L·mol^–1^·s^–1^]
0.074	333.15	0.25	70,800	129,100	-	16.5
0.049	333.15	0.25	70,200	127,900	193,900	16.2
0.074	333.15	0.5	41,300	72,800	111,900	18.7
0.049	333.15	0.5	41,100	72,500	110,000	18.5
0.074	323.15	0.25	61,800	115,200	-	14.6
0.049	323.15	0.25	61,200	113,500	-	14.4
0.074	323.15	0.5	33,930	61,600	-	15.8
0.049	323.15	0.5	35,000	61,000	-	16.0
0.074	313.15	0.25	47,200	92,800	-	11.4
0.049	313.15	0.25	50,500	93,400	136,800	11.6
0.074	313.15	0.5	26,200	49,800	-	12.5
0.049	313.15	0.5	28,400	48,400	-	12.8
0.049	303.15	0.5	19,400	39,100	-	9.5
0.074	293.15	0.25	12,100	22,400	32,700	2.8
0.049	293.15	0.25	12,400	20,700	32,100	2.7
0.074	293.15	0.5	16,100	29,600	-	7.5
0.049	293.15	0.5	14,300	30,100	-	7.1

**Table 5:** Experimental Arrhenius parameters for chain-length-averaged *k*_p_ of DnPI, obtained via PLP-SEC.

*A* [L·mol^–1^·s^–1^]	*E_A_* [kJ·mol^–1^]
(1.0 ± 0.5) · 10^4^	17.5 ± 1.2

**Table 6:** Comparison of Arrhenius prefactors and activation energies for $k_{p}$ of different itaconic acid esters. DMI = Dimethyl itaconate, DnBI = Di-n-butylitaconate and DCHI = Dicyclohexylitaconate.

Substance	*A* [10^5^ L·mol^–1^·s^–1^]	*E_A_* [kJ·mol^–1^]
DMI [6]	2.2	24.9
DMI [7]	7.3	27.8
DEI (this work)	0.11	17.5
DnPI (this work)	0.10	17.6
DnBI [7]	0.33	21.3
DCHI [7]	0.99	26.5
DCHI [8]	0.17	22.0

**Figure A1:**
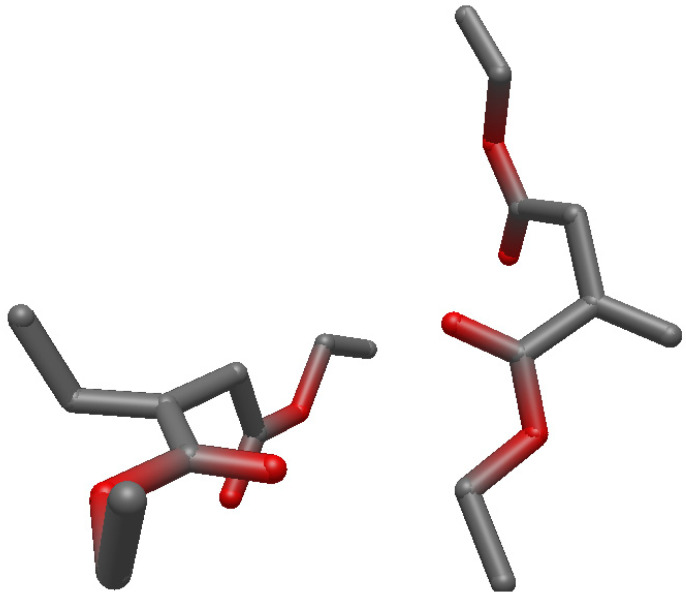
Structure of the reactants on UHF/6-31G(d) level of theory.

**Figure A2:**
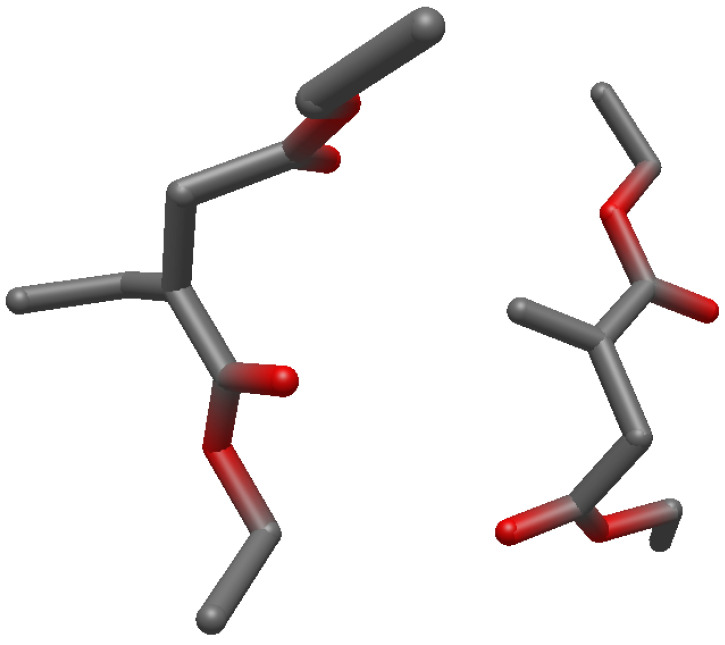
Structure of the transition state on UHF/6-31G(d) level of theory.

**Figure A3:**
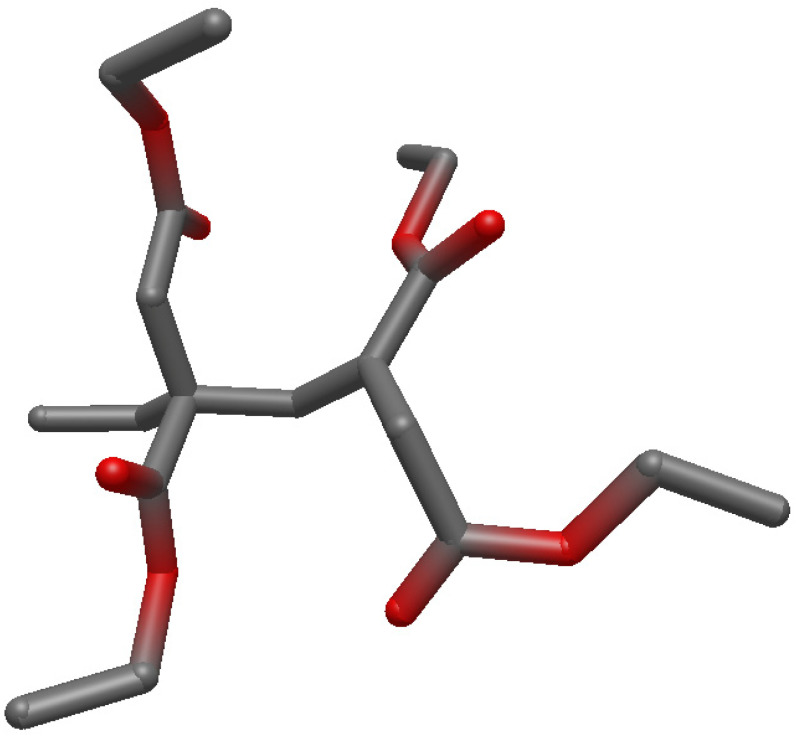
Structure of the product on UHF/6-31G(d) level of theory.

## Data Availability

The data presented in this study are available in this work.

## Appendix A

### Formulae for Calculating the Individual Entropy Components

$$S_{trans}=R\left(\ln\left({\left(\frac{2\pi Mk_{B}T}{h^{2}}\right)}^{\frac{3}{2}}\frac{k_{B}T}{p}\right)+1+\frac{3}{2}\right)$$

$$S_{rot}=R\left(\ln\left(\frac{\pi^{\frac{1}{2}}}{\sigma_{r}}\left(\frac{T^{\frac{3}{2}}}{\Theta_{r,x}\Theta_{r,y}\Theta_{r,z}}\right)\right)+\frac{3}{2}\right)$$

$$S_{vib}=R\underset{i}{\sum }\left(\frac{\frac{h\nu_{i}}{k_{B}T}}{\exp\left(\frac{h\nu_{i}}{k_{B}T}\right)-1}-\ln\left(1-\exp\left(\frac{h\nu_{i}}{k_{B}T}\right)\right)\right)$$

## Appendix B

### Structures of the Reactants, Transition State and Product on UHF/6-31g(d) Level of Theory

58

Reactants UHF/6-31G(d)

C -4.40972401531386 -3.71503806114475 -4.19211604628649

C -3.68133265382649 -2.50113964837613 -3.82846057294931

C -2.18555069312768 -2.58287955867867 -3.74895926164922

H -1.81956361353836 -1.90927486262867 -2.97994166704005

C -1.55123355560259 -2.21500407854842 -5.08428872443647

H -1.85981769088834 -3.58155274742932 -3.48373455760964

O -3.87993817666339 -4.76861766065154 -4.41207592473984

O -2.19867266439477 -2.02984556122397 -6.06256526758899

O -5.72557494763414 -3.54020745632567 -4.24515591923209

C -6.54017447797737 -4.64564347728663 -4.61958058930150

O -0.23107825111582 -2.11988533201135 -5.14587436878297

C 0.62860159593704 -2.24965352535422 -4.01615485238005

C -6.84381429855741 -5.54313533752586 -3.43397987468031

H -6.05382188123488 -5.19375753995276 -5.41217021980953

H -7.44408010230698 -4.19722006485559 -5.00595428455019

C 2.05126359981739 -2.21283246002602 -4.53134556977455

H 0.43228935067523 -3.17915492013272 -3.50505209750336

H 0.44776061679191 -1.42471687501624 -3.33574782757450

H -7.52779037380381 -6.33091526848121 -3.73515231333415

H -7.30921982574788 -4.97521989160534 -2.63537015695070

H -5.93929548340384 -6.00294157450675 -3.05616604113733

H 2.22599638910386 -3.03416206606115 -5.21539910098320

H 2.74329238575857 -2.31283602695059 -3.70356746094692

H 2.24579827549218 -1.28111638750133 -5.05035417824159

C 1.41023686313188 -7.39479693429179 -3.21670814323402

C 1.76218588985207 -7.00859229226371 -1.80537284102727

C 0.40030108509587 -6.50567479832831 -3.86586886936411

C 2.51335328251719 -5.68767506802261 -1.74174766720343

H 2.39075946646450 -7.77227607069698 -1.35759309022347

H 0.86155241563142 -6.94414051075366 -1.20845866996825

C 1.90936515216638 -8.44863568539955 -3.83552909442938

O 2.96604170334251 -5.15779889207647 -2.69980821647521

O -0.13492953713609 -5.62162351698555 -3.26298085202178

O 0.15592786208118 -6.79625093352468 -5.12018673812008

C -0.78815096521645 -5.98171011233685 -5.82956890531792

O 2.69679684031063 -5.13549053655022 -0.54522744993166

C 2.15076670664725 -5.65150973860652 0.65601647843522

C -0.99407495047473 -6.61281636474743 -7.18859925965537

H -0.37738582773625 -4.98579544880722 -5.91623978867437

H -1.70513446640562 -5.92923065237836 -5.26468399659097

C 2.67742937800976 -4.79518178786613 1.78889817293130

H 2.44840934259808 -6.68468532830934 0.79052106358879

H 1.06902617152998 -5.60801958172911 0.61572335119833

H -0.06041114415965 -6.66737370844859 -7.73713970390276

H -1.69456867264684 -6.01422677361150 -7.76061057091692

H -1.39699203958323 -7.61500923783392 -7.09171340373149

H 3.75936486793610 -4.83910902452768 1.82657350671995

H 2.38120460531527 -3.76220599922446 1.65136599612789

H 2.28125101852981 -5.14748058512089 2.73574103607832

C -4.37617783051305 -1.18068970392636 -3.63246950175260

H 2.62660274641475 -9.09041746684623 -3.35615376276326

H 1.62192414224581 -8.69793813042399 -4.83783698934764

C -4.86717639532277 -0.97403239860796 -2.19209531994862

H -5.21608532866781 -1.10596935176055 -4.31090971943407

H -3.68620846515025 -0.38550755085006 -3.89743691175879

H -5.59402029389089 -1.73139093368148 -1.91991355137942

H -5.33872079244902 -0.00206617048913 -2.08720830723050

H -4.04695233890537 -1.02343832869364 -1.48156140319029

58

TS UHF/6-31G(d)

C -2.84758329469564 -0.60649978656624 -0.35173091904958

C -3.47487656697929 0.67281650602152 -0.69218860128065

C -3.55472220027557 1.66082573684066 0.44012508768508

H -4.15851870941808 2.51469364647842 0.14752602094326

C -2.17076736081956 2.21218446327552 0.76780052421886

H -4.02699926242146 1.20732082219422 1.29793260123681

O -2.37853165100162 -0.81041029727699 0.73762180050786

O -1.41235165557832 2.49882171602563 -0.10039626506317

O -2.84933008160362 -1.48558407651005 -1.33176369020927

C -2.19960394673800 -2.74805070080274 -1.12824762851276

O -1.83173239905996 2.43331079819363 2.02868690080066

C -2.55087438021810 1.92566714844963 3.14350614153460

C -3.11843172362171 -3.73239536663273 -0.43066657627182

H -1.28476954398572 -2.59602904116585 -0.58112513653719

H -1.95694673813358 -3.08261012013724 -2.12545612828486

C -1.74871064876934 2.27094685075197 4.38067005218506

H -2.66491389394941 0.85539962284188 3.04179449838902

H -3.53222003290299 2.38406925600524 3.18726109676217

H -2.62772884930282 -4.69814852441827 -0.36306955244555

H -4.04290923096498 -3.85660274198890 -0.98517311104905

H -3.35315580445981 -3.40034850572737 0.57317343240027

H -0.76717665857079 1.81482615852842 4.33393178450831

H -2.25947451406035 1.90665701863723 5.26621241567900

H -1.62511979519397 3.34401516574444 4.46827123098726

C 2.45871053755559 -0.26177323025727 0.56762667061529

C 2.79559014608955 -1.71516497412086 0.87362760554040

C 3.61166865724519 0.64959838060042 0.31640811668741

C 2.36276327964106 -2.64269924413079 -0.25522296185348

H 2.22573018964650 -2.03420907136342 1.73606841408987

H 3.84303814407620 -1.80421504547035 1.10979341341543

C 1.21370556363695 0.18845387067221 0.53453341572562

O 1.22302307625872 -2.95947287516606 -0.37567425946026

O 4.75138414735338 0.28560266220910 0.31494859834329

O 3.25990506702815 1.90066757854423 0.09691365205411

C 4.29380168181435 2.84598959018137 -0.15811209860918

O 3.25092318791082 -3.09808863238834 -1.12612844328230

C 4.65135217212255 -2.86463776788425 -1.03645456782551

C 3.63156516776283 4.19033256304175 -0.37090343771677

H 4.85059252285008 2.53064888670376 -1.02987695753387

H 4.97175416491287 2.86083895217022 0.68409712423064

C 5.27640883314746 -3.46428606983801 -2.27856471421145

H 5.03901151894821 -3.34541579398755 -0.14555211646049

H 4.85187306822269 -1.80671745273000 -0.96978488472731

H 2.94832090725509 4.15365783576250 -1.21135921475387

H 4.38626883919165 4.94414590789859 -0.57156183280494

H 3.07299069277300 4.48727374435213 0.50915042355221

H 5.06662336133763 -4.52580732703888 -2.33870880032320

H 6.35211780068496 -3.32282494115468 -2.25478158261550

H 4.88443282638933 -2.98689481786365 -3.16908667469771

C -3.97084269224552 1.00933932810088 -2.06935207777060

H 0.38495916637729 -0.47045358745645 0.71628589321382

H 0.98343794180324 1.21339663085629 0.32059547184296

C -5.47876335273979 0.76682926161363 -2.23434806872066

H -3.43270737829562 0.42171878332917 -2.80110921489571

H -3.74481810647014 2.05270880870212 -2.26764947487017

H -5.71983714072145 -0.27989805219422 -2.08236802290827

H -5.80030204259206 1.04437265979783 -3.23290135138030

H -6.05723300624604 1.35210768974649 -1.52524402102384

58

Product UHF/6-31G(d)

C -5.33010044431225 -5.17468805041027 -2.76289465235525

C -4.78315772241794 -3.74187542846288 -2.83238392739415

C -4.91490745292611 -3.16240216496752 -1.40859455783708

H -5.94684554863516 -3.24388163191316 -1.08693595720722

C -4.49533849687114 -1.71842485435190 -1.20853512882611

H -4.36388407521240 -3.78964728582604 -0.72205388163776

O -5.58232282598512 -5.74716981209004 -1.74444107985903

O -4.23777261302297 -0.96449508470472 -2.08821498403281

O -5.47736666409740 -5.70975276825688 -3.95247798831326

C -5.84141129580070 -7.08827845545773 -4.05745733149766

O -4.44462443011922 -1.26384739619001 0.04233381138976

C -4.68964894858728 -2.02009241215792 1.21933639715133

C -7.34751799177768 -7.26186776811452 -4.01977300066089

H -5.35587379264847 -7.63996552105348 -3.26963765395188

H -5.42893426724061 -7.40053252050025 -5.00366130343885

C -3.39021667860755 -2.51346724885203 1.82991718662646

H -5.37280836558159 -2.83407224254007 1.02766841874399

H -5.18154748745600 -1.33074205897361 1.89050822281558

H -7.59816478269545 -8.30664551845971 -4.17670802357371

H -7.82162518208360 -6.67825133235716 -4.80173076367217

H -7.74968835253830 -6.95655894609922 -3.06141754447941

H -2.86885690800861 -3.20089536207003 1.17303179101418

H -3.59294050548452 -3.02702278893814 2.76445031994558

H -2.73133992171486 -1.67742526636710 2.03373806871067

C -2.25638271644363 -4.29802356482885 -2.34672033075670

C -2.14994288261521 -5.75792961322905 -1.95614587436087

C -1.27560572228266 -3.38516847385001 -1.73135212323383

C -1.74501007255527 -6.62518498669894 -3.14442466679776

H -3.11702183256505 -6.12824495221061 -1.64444855005516

H -1.47069622074738 -5.85160883143027 -1.12546239144077

C -3.29627120196457 -3.79678101600397 -3.32477710297106

O -2.55018701171806 -6.97278116004941 -3.94624601760557

O -0.52009825073052 -3.72531508379593 -0.86073931989745

O -1.30784908742213 -2.16235825899936 -2.21815076002935

C -0.45753853908387 -1.17878825589158 -1.63106437802248

O -0.47828520965378 -6.97610937240425 -3.30670509988317

C 0.55342894625031 -6.71420088237352 -2.36305862143452

C -0.72664336509408 0.12928609417126 -2.34257114085629

H 0.57036870257388 -1.49752056363488 -1.73829056138870

H -0.68133578016605 -1.11308157749710 -0.57537710329629

C 1.84663386032022 -7.20646861946591 -2.97771857588070

H 0.34209716384925 -7.24440752572095 -1.44200542427960

H 0.60382294400404 -5.65799792058409 -2.14495476578319

H -0.49099363644908 0.04715871551529 -3.39782889373174

H -0.10843043217604 0.91170677765523 -1.91371063533207

H -1.76790844150780 0.40723082368249 -2.24144803072511

H 1.78662780732825 -8.26617024389027 -3.19551251175610

H 2.66870361910261 -7.03821915779318 -2.28974090521574

H 2.05389412046677 -6.67831874528678 -3.90101482203190

C -5.59021265597694 -2.95347734228697 -3.89736483461998

H -3.27283361976153 -4.42357249879311 -4.20844199940660

H -3.02628626467457 -2.79692449756745 -3.62351497206030

C -7.08732575940939 -2.81039347414719 -3.61393750029032

H -5.45801438274024 -3.45658708075387 -4.84652279696569

H -5.15327409048206 -1.97142361922166 -3.98813513854066

H -7.57585224399955 -3.76960956349807 -3.47008243545291

H -7.57226269412077 -2.32368534091258 -4.45365716857534

H -7.27933029572944 -2.19952826908910 -2.73799098498188

## References

[1] Panic V.V., Seslija S.I., Popovic I.G., Spasojevic V.D., Popovic A.R., Nikolic V.B., Spasojevic P.M. (2017). Simple One-Pot Synthesis of Fully Biobased Unsaturated Polyester Resins Based on Itaconic Acid. Biomacromolecules.

[2] Dai Z., Yang Z., Chen Z., Zhao Z., Lou Y., Zhang Y., Liu T., Fu F., Fu Y., Liu X. (2018). Fully Biobased Composites of an Itaconic Acid Derived Unsaturated Polyester Reinforced with Cotton Fabrics. ACS Sustain. Chem. Eng..

[3] Wierckx N., Agrimi G., Lübeck P.S., Steiger M.G., Mira N.P., Punt P.J. (2020). Metabolic specialization in itaconic acid production: A tale of two fungi. COBIOT.

[4] Zhao M., Lu X., Zong H., Li J., Zhuge B. (2018). Itaconic acid production in microorganisms. Biotechnol. Lett..

[5] Steiger M.G., Blumhoff M.L., Mattanovich D., Sauer M. (2013). Biochemistry of microbial itaconic acid production. Front. Biomol..

[6] Yee L.H., Coote M.L., Chaplin R.P., Davis T.P. (2000). Determination of propagation rate coefficients for an α-substituted acrylic ester: Pulsed laser polymerization of dimethyl itaconate. J. Polym. Sci. A Polym. Chem..

[7] Szablan Z., Stenzel M.H., Davis T.P., Barner L., Barner-Kowollik C. (2005). Depropagation Kinetics of Sterically Demanding Monomers:  A Pulsed Laser Size Exclusion Chromatography Study. Macromolecules.

[8] Vana P., Yee L.H., Davis T.P. (2002). Multipulse Initiation in Pulsed Laser and Quenched Instationary Polymerization:  Determination of the Propagation and Termination Rate Coefficients for Dicyclohexyl Itaconate Polymerization. Macromolecules.

[9] Sato T., Inui S., Tanaka H., Ota T., Kamachi M., Tanaka K. (1987). Kinetic and ESR studies on the radical polymerization of Di-n-butyl itaconate in benzene. J. Polym. Sci. A Polym. Chem..

[10] Sato T., Takahashi Y., Seno M., Nakamura H., Tanaka H., Ota T. (1991). Effect of alkyl groups on the rate constants of propagation and termination in the radical polymerization of dialkyl itaconates. Die Makromol. Chem..

[11] Otsu T., Yamagishi K., Yoshioka M. (1992). Determination of absolute rate constants for radical polymerization of dialkyl itaconates with various ester groups by electron spin resonance spectroscopy. Macromolecules.

[12] Otsu T., Yamagishi K., Matsumoto A., Yoshioka M., Watanabe H. (1993). Effect of .alpha.- and .beta.-ester alkyl groups on the propagation and termination rate constants for radical polymerization of dialkyl itaconates. Macromolecules.

[13] Sato T., Hirose Y., Seno M., Tanaka H., Uchiumi N., Matsumoto M. (1994). Kinetic and ESR studies on radical polymerization. Radical polymerization of diisopropyl itaconate. Eur. Polym. J..

[14] Becker H. (2001). Organikum: Organisch-Chemisches Grundpraktikum.

[15] Veličković J., Vasović S. (1972). KUHN-MARK-HOUWINK-SAKURADA relations and unperturbed dimensions of poly(di-n-alkyl itaconates). Die Makromol. Chem..

[16] Pracht P., Bohle F., Grimme S. (2020). Automated exploration of the low-energy chemical space with fast quantum chemical methods. Phys. Chem. Chem. Phys..

[17] Neese F. (2012). The ORCA program system. Wiley Interdiscip. Rev. Comput. Mol. Sci..

[18] Neese F. (2018). Software update: The ORCA program system, version 4.0. Wiley Interdiscip. Rev. Comput. Mol. Sci..

[19] Beuermann S., Harrisson S., Hutchinson R.A., Junkers T., Russell G.T. (2022). Update and critical reanalysis of IUPAC benchmark propagation rate coefficient data. Polym. Chem..

[20] Beuermann S., Buback M. (2002). Rate coefficients of free-radical polymerization deduced from pulsed laser experiments. Prog. Polym. Sci..

[21] Coote M.L., Mark H.F. (2006). Computational Quantum Chemistry For Free-Radical Polymerization. Encyclopedia of Polymer Science and Technology.

[22] Edeleva M., Van Steenberge P.M.H., Sabbe K.M., D’hooge D.R. (2021). Connecting Gas-Phase Computational Chemistry to Condensed Phase Kinetic Modeling: The State-of-the-Art. Polymers.

[23] Scott A.P., Radom L. (1996). Harmonic Vibrational Frequencies:  An Evaluation of Hartree−Fock, Møller−Plesset, Quadratic Configuration Interaction, Density Functional Theory, and Semiempirical Scale Factors. J. Phys. Chem..

[24] Kesharwani M.K., Brauer B., Martin J.M.L. (2015). Frequency and Zero-Point Vibrational Energy Scale Factors for Double-Hybrid Density Functionals (and Other Selected Methods): Can Anharmonic Force Fields Be Avoided?. J. Phys. Chem. A.

[25] Nitschke A., Riemann L., Kollenbach L., Braun V., Buback M., Vana P. (2020). Investigation into the Kinetics of n-Pentyl Methacrylate Radical Polymerization. Macromol. Chem. Phys..

[26] Olaj O.F., Bitai I., Hinkelmann F. (1987). The laser-flash-initiated polymerization as a tool of evaluating (individual) kinetic constants of free-radical polymerization, 2. The direct determination of the rate of constant of chain propagation. Die Makromol. Chem..

[27] Olaj O.F., Vana P., Zoder M., Kornherr A., Zifferer G. (2000). Is the rate constant of chain propagation kp in radical polymerization really chain-length independent?. Macromol. Rapid Commun..

[28] Olaj O.F., Vana P., Zoder M. (2002). Chain Length Dependent Propagation Rate Coefficient kp in Pulsed-Laser Polymerization:  Variation with Temperature in the Bulk Polymerization of Styrene and Methyl Methacrylate. Macromolecules.

[29] Olaj O.F., Zoder M., Vana P., Kornherr A., Schnöll-Bitai I., Zifferer G. (2005). Chain Length Dependence of Chain Propagation Revisited. Macromolecules.

[30] Nikitin A.N., Dušička E., Lacík I., Hutchinson R.A. (2022). Chain-length dependence of the propagation rate coefficient for methyl acrylate polymerization at 25 °C investigated by the PLP-SEC method. Polym. Chem..

[31] Gridnev A.A., Ittel S.D. (1996). Dependence of Free-Radical Propagation Rate Constants on the Degree of Polymerization. Macromolecules.

[32] Heuts J.P.A., Russell G.T. (2006). The nature of the chain-length dependence of the propagation rate coefficient and its effect on the kinetics of free-radical polymerization. 1. Small-molecule studies. Eur. Polym. J..

[33] Willemse R.X.E., Staal B.B.P., van Herk A.M., Pierik S.C.J., Klumperman B. (2003). Application of Matrix-Assisted Laser Desorption Ionization Time-of-Flight Mass Spectrometry in Pulsed Laser Polymerization. Chain-Length-Dependent Propagation Rate Coefficients at High Molecular Weight:  An Artifact Caused by Band Broadening in Size Exclusion Chromatography?. Macromolecules.

[34] Beuermann S. (2002). Requirements Associated with Studies into a Chain-Length Dependence of Propagation Rate Coefficients via PLP−SEC Experiments. Macromolecules.

[35] Haehnel A.P., Schneider-Baumann M., Hiltebrandt K.U., Misske A.M., Barner-Kowollik C. (2013). Global Trends for kp? Expanding the Frontier of Ester Side Chain Topography in Acrylates and Methacrylates. Macromolecules.

